# Supplementary material for: Strengthening systems to provide long-acting reversible contraceptives (LARCs) in public sector health facilities in Uganda and Zambia: Program results and learnings
Source: PLoS One. 2023 Aug 18;18(8):e0290115. doi: 10.1371/journal.pone.0290115 (PMC10437908; doi:10.1371/journal.pone.0290115)
Supplement: S1 Table — This table includes the LARC-related activities implemented by government and CHAI in Uganda and Zambia in this program. (DOCX) [file pone.0290115.s001.docx]

**S1 Table. Key LARC-related Program Activities.** This table includes the LARC-related activities implemented by government and CHAI in Uganda and Zambia in this program.

| **SRMNH Program Pillar** | **Key LARC-related activities in Uganda** | **Key LARC-related activities in Zambia** |
| --- | --- | --- |
| **LARC training and mentorship to build skills and confidence of health workers** | - Collaborated with Association of Obstetricians and Gynecologists of Uganda (AOGU) to deploy in-service training on LARC to 217 public sector health workers across 85 facilities - Activated national, regional and district government clinical mentorship structures to deploy integrated SRMNH mentorship to focus facilities, in partnership with AOGU - Deployed LARC mentorship immediately after trainings and again as needed, based on mentors’ review of health worker competency and service data and needs identified by facility and district leadership - Printed and distributed job aids, checklists and training materials on family planning and LARCs - Integrated LARC skills and supplies checklists into clinical mentorship tools for routine use during government-led health worker mentoring and Quality Improvement (QI) activities - Improved content of group health education during antenatal care (ANC) and postnatal care (PNC) sessions to reinforce correct information on LARC benefits and side effects - Mobilized clients ahead of health worker training to ensure sufficient opportunities for health workers to gain practice and supports skills development - Worked with community health workers (Village Health Teams) to routinize family planning counselling and provision of short-term contraceptive methods in communities and referrals onwards to facilities for LARC services | - Collaborated with Zambia Association for Gynaecologists and Obstetricians (ZAGO) to deploy LARC in-service training to 146 public sector health workers - Operationalized national mentorship guidelines by scaling electronic family planning mentorship tools and dashboards - Expanded pool of subnational government mentors and provided capacity building on LARC skills and mentorship - Supported deployment of LARC mentorship utilizing government mentors, institutionalizing reoccurring opportunities for demonstration and practice with anatomical models and clients, to foster retention of skills - Supported updates to FP counselling tools to help health workers address myths and misconceptions and provide information on LARC side effects management - Printed and distributed job aids, checklists and training manuals on FP and LARCs - Developed and tested cost-efficient models for LARC training, mentorship, and community-based distributor training; facilitated adoption of models by national FP program for replication across additional geographies. - Increased awareness of postabortion family planning guidelines by disseminating national Comprehensive Abortion Care (CAC) guidelines to facilities - Integrated new LARC methods into the national Essential Medicine List (EML), essential guidelines used by facilities to guide service delivery - Worked with community-based distributors (CBDs), a government-approved volunteer cadre, to routinize family planning counseling and provision of short-term contraceptive methods in communities and referrals onwards to facilities for LARC services - Encouraged peer educators to routinize family planning referrals from schools and communities to improve awareness and access among adolescents and reach in-school and out-of-school youth |
| **Supply chain strengthening to ensure the availability of reliable and affordable commodities, consumables and equipment for key services** | - Used program funding and results-based financing to procure implant and IUD insertion and removal equipment to fill immediate gaps - Improved methodology of annual procurement planning via government District Medicines Management Supervisors to enable public sector procurement and distribution entity National Medical Stores to meet facility supply needs - Procured implant and IUD insertion and removal equipment to fill immediate gaps, in absence of available domestic resources - Developed and deployed district supply chain dashboards for visualization of health facility stock data to inform emergency ordering and intra-district commodity redistribution - Activated low-cost communication channels using messaging applications for efficiency in flagging stock issues and redistribution needs to District Medicines Management Supervisors | - Used program funding to procure implant and IUD insertion and removal equipment to fill immediate gaps, in absence of available domestic resources - Strengthened national and provincial forecasting and quantification meetings to ensure availability of FP and other RH commodities - Developed IUD equipment quantification to support domestic resource allocation and resource mobilization to fill equipment gaps - Supported redistribution of FP commodities within and across provinces to address stockouts in districts - Routinized monitoring of LARC supplies |
| **Demand generation outreach to sensitize the community** | - Collaborated with community radio stations to feature district and community leaders and health workers as talk show panelists to promote local public sector facilities as access points for high quality contraceptive services - Supported parish and village chiefs to share information on benefits of healthy child spacing, and on how LARC methods can be used to achieve family planning goals within their communities - Ensured that routine clinical outreach led by facility staff integrated LARC service provision to expand to lower-level health facilities in catchment zones - Created awareness of sexual health service benefits amongst adolescents by establishing referral pathways and outreach channels between schools and local health facilities | - Added information on LARC service options and benefits within the national government Community Health Assistants (CHA) training curriculum to increase access to information on LARC services within communities - Supported broader FP demand creation and trainings for CBDs to promote FP options awareness - Fostered buy-in with traditional leaders, working with cultural gate keepers to promote the benefits of FP within their spheres of influence and address common local myths and misconceptions of LARC - Ensured that routine clinical outreach led by facility staff integrated LARC services to expand access to information and services to hard-to-reach communities in lower-level health posts in catchment zones - Created demand for contraception amongst adolescents by training peer educators to conduct mobilization activities among adolescents at schools and in communities |
| **Cross-cutting: management and information systems strengthening to support service delivery and data tracking** | - Institutionalized health information management system data analysis and use of Reproductive Maternal Newborn Child and Adolescent Health score cards as part of performance management practices within local administrative standard operating procedures | - Developed and integrated age-disaggregated family planning consumption indicators into the national health management information system (HMIS), enabling regular review of FP uptake among adolescents and subsequent program action - Routinized sharing family planning data insights with the national Family Planning Technical Working Group (TWG) to improve visibility into performance and challenges, and formulate programmatic action to support facilities in Northern Province - Integrated new FP methods into HMIS and FP register to improve FP tracking - Improved performance management by triangulating service provision, training and mentorship provision, and commodity availability data at facilities, thereby driving programmatic follow-up to address facility gaps |
