# Supplementary material for: Strengthening systems to provide long-acting reversible contraceptives (LARCs) in public sector health facilities in Uganda and Zambia: Program results and learnings
Source: PLoS One. 2023 Aug 18;18(8):e0290115. doi: 10.1371/journal.pone.0290115 (PMC10437908; doi:10.1371/journal.pone.0290115)
Supplement: Attachment — Submitted filename: Reviewer.pdf [file pone.0290115.s004.pdf]

Strengthening systems to provide Long-Acting Reversible Contraceptives (LARCs) in public sector health facilities in Uganda and Zambia: Program results and learnings.

Summary.

The authors conducted a study looking at strengthening systems to provide Long-Acting Reversible Contraceptives (LARCs) in public sector health facilities in Uganda and Zambia: Program results and learnings.

This study is significant and applicable to both study settings as there is a high rate of unintended pregnancies. The article is generally well written, but the following points need to be considered:

1. The contextual differences or similarities between the two countries/study sites should be explained. This is crucial because it will enable a better understanding of the variations in project intervention uptake, such as the use of intrauterine devices (IUDs). The discussion's elaborated contextual explanations are insufficient. The authors might also consider other social and cultural aspects that have been covered in other works of literature.

2. The authors ought to explain why particular provinces in the two countries were chosen. How does the provision of sexual, reproductive, maternal, and new-born health (SRMNH) in each nation compare on a provincial level?

3. The authors must describe the type/category of health professionals trained at these facilities. Discuss how the project dealt with staff attrition throughout the course of the study as well.

Minor comments

1. Provide a reference for Figure 1. Virtuous cycle of LARC availability and provision.

2. In the discussion section first paragraph "In Zambia, Marie Stopes Zambia was active in Zambian program geographies, and at the time of baseline data collection had recently started reporting into public sector facility registers." Rephrase to avoid text redundancy
